# A few of our favorite unconfirmed ideas

**DOI:** 10.1186/cc14719

**Published:** 2015-12-18

**Authors:** John J Marini, Luciano Gattinoni, Can Ince, Sibylle Kozek-Langenecker, Ravindra L Mehta, Claude Pichard, Martin Westphal, Paul Wischmeyer, Jean-Louis Vincent

**Affiliations:** 1 University of Minnesota, Minneapolis/St. Paul, MN, USA; 2 Dipartimento di Fisiopatologica Medico-Chirurgica e dei Trapianti, Università degli Studi di Milano, Milan, Italy; 3 Dipartimento di Anestesia, Rianimazione ed Emergenza Urgenza, Fondazione IRCCS Ca' Granda--Ospedale Maggiore Policlinico di Milano, Milan, Italy; 4 Department of Intensive Care Medicine, Erasmus Medical Center, Rotterdam, the Netherlands; 5 Sigmund Freud Private University, Vienna, Austria; 6 Department of Anesthesia and Intensive Care, Evangelical Hospital Vienna, Hans Sachs-Gasse Vienna, Austria; 7 Department of Medicine, University of California San Diego, San Diego, CA, USA; 8 Department of Clinical Nutrition, Geneva University Hospital, Geneva, Switzerland; 9 Department of Anesthesiology, Intensive Care and Pain Medicine, University of Muenster, Muenster, Germany; 10 Fresenius Kabi AG, Bad Homburg, Germany; 11 Department of Anesthesiology, University of Colorado, Aurora, Colorado; 12 Department of Intensive Care, Erasme University Hospital, Université libre de Bruxelles, Brussels, Belgium

## Abstract

Medical practice is rooted in our dependence on the best available evidence from incremental scientific experimentation and rigorous clinical trials. Progress toward determining the true worth of ongoing practice or suggested innovations can be glacially slow when we insist on following the stepwise scientific pathway, and a prevailing but imperfect paradigm often proves difficult to challenge. Yet most experienced clinicians and clinical scientists harbor strong thoughts about how care could or should be improved, even if the existing evidence base is thin or lacking. One of our Future of Critical Care Medicine conference sessions encouraged sharing of novel ideas, each presented with what the speaker considers a defensible rationale. Our intent was to stimulate insightful thinking and free interchange, and perhaps to point in new directions toward lines of innovative theory and improved care of the critically ill. In what follows, a brief background outlines the rationale for each novel and deliberately provocative unconfirmed idea endorsed by the presenter.

## Introduction

With good justification, medicine is generally viewed as a conservative discipline whose practice changes at a deliberate pace. To some extent, this caution is rooted in our dependence on the published results of incremental scientific experimentation and rigorous clinical trials to determine the worth of ongoing practice and/or to guide innovation. In many respects, such a careful approach serves us well. But progress made in this way can be slow, and a prevailing but imperfect paradigm often proves difficult to challenge. Yet most experienced clinicians and clinical scientists harbor strong thoughts about how care could or should be improved, even if the existing evidence base is thin or lacking. One of our Future of Critical Care Medicine (FCCM) conference sessions encouraged the sharing of such ideas, each presented with what the speaker considers a defensible (if unverified) rationale. The intent of this provocative format was to stimulate thinking and free interchange, and perhaps to point in new directions toward lines of innovative theory and improved care of the critically ill. Of the eight presentations, two can be considered to address ways to improve the current processes of care delivery, two to refine existing mechanical organ support methodologies, two to better assess, regulate and time treatment options, and two to utilize nutritional pathways to improve patient response to life-threatening illness and reduce the risk of chronic critical illness. In what follows, a brief background outlines the rationale for each novel and deliberately provocative unconfirmed idea endorsed by the presenter.

## Promote passion and laughter to energize team spirit and improve performance

Martin Westphal, MD, PhD

### Background

**Figure 1:**
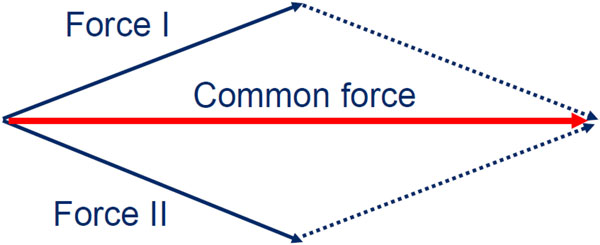
**Synergy of forces acting with common purpose**.

Passion and humor not only represent "nice to have" features of the caregiving environment, but also important contributors to performance and positive results. Respectful cooperation and communication among staff members of the multidisciplinary team are fundamental to effective management and good patient outcomes. In this context, the attending physician (the supervisor) should provide professional teaching and encourage the maturation of the residents, interns, and students. In turn, the residents should express respect for their supervisors, thereby contributing to a positive feedback loop and culture. The why, when, and how of decision-making should be convincingly explained whenever possible. In settings characterized by lack of mutual respect and cooperation, complaints abound and work is done simply because of necessity or the obligations of duty, but not from conviction. Team leaders should always target connection among team members and focus their attention on the common goal of patient recovery (Figure 1). True team spirit cannot be forced by monetary incentives and/or threats, but rather by passion and humor created within and helping to nurture a positive working environment. That humor and laughter are important not only for staff members but also for certain patient populations has been demonstrated in the literature [1,2]. Working as a team increases satisfaction and performance of team members. Passion for the subject and humor represent key features that encourage team spirit.

### Idea

Respect, humor, and positive attitudes build the team spirit that makes highly effective critical care possible.

## Target outcome quality indicators in perioperative intensive care: goal-directed pro-action trumps the need for re-action

Sibylle Kozek-Langenecker, MD

### Background

Much of critical care practice is based upon reaction to clinical signs and laboratory findings. As a consequence, the physician is often one step behind the pathological process. In perioperative critical care the consequences of this approach may be avoidable postoperative complications that delay recovery or impair long-term survival after major surgery. A newer approach is to institute goal-directed therapy based on optimized flow parameters. Improved tissue perfusion by targeting a stroke volume optimized to flow (with stopping rules) is a promising modern alternative to conventional pressure-targeted approaches in this arena. In some recent studies, complications have been reduced dramatically using colloids and crystalloids in preference to transfused red blood cells. In fact, after major surgery the administration of unneeded hemoglobin has been associated with increases in ventilation time, septicemia, pneumonia, and renal failure, among other complications. Threshold concentrations of hemoglobin once thought appropriate now appear to have been set too high for most patients. Revision of the transfusion threshold to lower values appears judicious unless such signs as ST segment elevation, tachycardia, hypotension, or lactic acidosis prompt an exception to be made.

### Idea

Monitoring quality indicators of outcome routinely, with evaluation by benchmarks and peer reviews, will allow us to both realize and quantify real-life improvements in perioperative intensive care. Flow-guided volume therapy is often essential to achieve sustained net clinical benefit in the perioperative setting.

## Limit power delivery as well as airway pressures to avoid ventilator-associated lung injury

Luciano Gattinoni, MD

### Background

Although excessive forces applied to the lung during mechanical ventilation are known to cause lung damage, the exact mechanism by which these physical forces cause injury and therefore the hazard targets and thresholds at which the clinician should direct attention are less well understood. Experiments have shown that damage occurs when tidal volume encroaches upon the total lung capacity and pressures rise into a dangerous zone. The total lung capacity of the injured baby lung varies from patient to patient, and therefore tidal volume represents an insufficient marker of ventilator-induced lung injury (VILI) risk when considered alone without knowing the aerated volume into which it is delivered. In fact, what appears to hurt the lung structure more closely relates to the energy load that the tissue sustains [3]. The determining variables of the delivered energy per unit time (power) are the peak pressure and excursion of pressure which the tidal volume generates within the baby lung, the flow rate (how fast the tidal volume is delivered and pressure builds), and the respiration frequency, an intuitively important variable to consider, whatever the characteristics of the individual tidal cycle might be. According to current understanding rooted in experimental science, we need to become more sophisticated in our understanding of the variables to control and the targets to identify. A key objective of ongoing work is to determine the power threshold of ventilation below which ventilation is always innocent and above which it is always dangerous. The quantification of such a threshold, an objective with firm biologic plausibility, will dictate the mandate to apply or withhold artificial extracorporeal support.

### Idea

Power delivered to the lung, rather than tidal volume or any specific pressure associated with the tidal volume, most directly determines the potential for VILI. Identification of the numerical threshold for power delivery that separates safety from injury is feasible to identify for clinical practice.

## Outsource organ support: the case for resting the kidney during critical illness

Ravindra L. Mehta, MD

### Background

Critical illness imposes a significant demand on all organ systems, including the kidney. The underlying disease process--for example, sepsis or burns--increases the metabolic load while the processes of care--for example, fluid resuscitation and nutritional support--contribute to salt and water retention and add a volume stress. In these settings the kidney has to work harder to meet the demand and relies on recruiting additional excretory capacity. Unfortunately, the available reserve capacity maybe compromised due to age, prior kidney disease, therapeutic interventions, or comorbidities. Furthermore, the kidney may be subject to impaired autoregulation from medications, cellular edema, and tubular damage from nephrotoxic agents.

Although resting the kidney in times of increased demand may seem counterintuitive, it is possible that the high demands of critical illness can be met equally well with continuous renal replacement therapy (CRRT) [4], thereby reducing the workload otherwise imposed on the kidney [5]. Moreover, the ability to dissociate solute clearance from volume management provides great flexibility in maintaining solute levels at any concentration without compromising volume status. Specific targets and goals can be met with the increased clearance capacity added to the kidney's own. To apply CRRT as an adjunctive, rather than a rescue measure, we will need to establish parameters that anticipate increased workload on kidney function and develop quantifiable metrics for monitoring dynamic changes in the demand and renal functional capacity [6].

### Idea

We should consider prophylactic intervention with CRRT as part and parcel of the strategy to manage ICU patients at high risk for renal functional compromise. We can identify these patients based on quantifiable criteria for demand and renal functional capacity. Preemptive CRRT for protecting kidney is a viable strategy that will need to be tested in prospective clinical trials.

## Use probiotics, poop pills, and fecal transplants to modify the gut microbiome to improve outcome?

Paul Wischmeyer, MD

### Background

The microbes which populate the gut are increasingly recognized to play important roles in the well-being of healthy adults. Billions of microbes (in varied proportions for different individuals) are found within the intestine, and the absorbed products of their activity influence the health of our patients. Many microbes of importance cannot be easily cultured, but almost all can be sequenced by their RNA. Taken together, this "microbiome" has a diverse genetic makeup with a profusion of genes that stands ready to improve or impair the host's physiological status. Different body habitats are very different from one another. Predictably, antibiotics strongly influence the composition of the microbiome. Indeed, the precise makeup for any individual is influenced by the environment in which he/she is immersed. Differences in the microbiome may be responsible for such phenotypic expressions as obesity versus normal habitus. Although a portion of that effect relates to the efficiency with which food is processed in the intestine, signals generated within the microbiome may be transported into the host, influencing hormonal conditions that prompt satiety or hunger. Given the potential power and diversity of the microbiome and our ability to manipulate its composition, it may be possible to manipulate the microbiome so as to combat disease and promote healing. The potential for this therapeutic approach has been recently exemplified in the elimination of *Clostridium difficile *intestinal infection by instillation of a fecal transplant into the colon of a well-prepared recipient [7]. Probiotics can influence the composition of the existing microbiome, often to the advantage of the patient, with recent systematic reviews showing a >60% reduction in *C. difficile *diarrhea post probiotic therapy [8]. Further, multiple trials of probiotics now demonstrate significant reductions in overall infections, including ventilator-associated pneumonia in the ICU [9,10]. The possibility of reconstituting the microbiome by first eliminating the ineffective bacterial populations and replacing them with beneficial ones tailored to improve patient status and combat disease would appear a promising prospect for the future. Progress has already been made in defining the microbiomes of health and critical illness. It remains to be determined which bacteria should be orally ingested or rectally instilled to target specific objectives. At this point it is clear that the provision of intensive care leads to significant loss in diversity of the gut microbiome and that this narrowing of the spectrum may be associated with worse outcome [11].

### Idea

We should utilize probiotics and "poop" pills and perform targeted stool transplants to re-sod the microbiome in the ICU to improve outcome.

## Optimize feeding of both the intestinal microbiome and the patient: dual (enteral-parenteral) nutritional support

Claude Pichard, MD, PhD

### Background

The importance of the gut microbiome to the well-being of the critically ill patient has been well demonstrated and described [12]. This microbiota represents an important and heretofore unrecognized "organ" which exerts great influence on a number of cellular mechanisms, such as those related to immunoreactivity, inflammation, energy homeostasis, etc. The microbiome is massively and negatively influenced during the ICU stay by many factors, which include antimicrobial treatments, exposure to pathogenic flora, fasting or poor nutrition and nonphysiologic sterile feedings, colonic stasis secondary to physical immobilization, and opioid analgesics [13]. The exact consequences of this radical shift in microbial composition are only now being explored. From all indications, however, it appears as if the door has been opened to understand the consequences of our current nutritional practices and of the potential for microbiome modification as a novel means by which to promote health and accelerate healing.

While stool transplantation has grabbed medical headlines and has been successfully applied outside the ICU setting in patients with refractory *C. diff*icile colitis, Crohn's disease, and severe obesity, it seems unlikely to be as easily applied to intensive care patients for reasons closely related to the timing of treatment and to the practical aspects of doing so in the ICU setting. To leverage this intriguing concept of microbiome manipulation, the approach must be modified for these constraints. In the initial phase of illness the goal is to maintain a diverse and physiologic mass of beneficial bacteria and yeasts. This will require administration of a feeding product which--far from being sterile--is mostly a mixture of physiologic bacteria and yeasts and their preferred food. Following that first phase, the goal will be to optimize this revised gut microbiome. To do so will require highly modified feedings that are designed primarily to nourish the microbes and secondarily to provide nutrients for the host. In such a setting, enteral nutrition aims at feeding primarily the intestinal microbiome, whereas parenteral nutrition could be used to provide or supplement the nutritional needs of the patient-host.

### Idea

Administer feedings that generate and promote a salutary intestinal microbiome for the critically ill patient.

## Dance in the rain: restrain dysfunctional responses to stress and promote gradual accommodation to abnormal physiologic targets

John J. Marini, MD

### Background

In its early years, the practice of critical care evolved from anesthetic approaches and postoperative practices. The natural inclination was to strive to restore the "normal" baseline. The high price of achieving and maintaining normality, however, has been to inflict unintended injury by our treatments, as exemplified by VILI, drug toxicities, and oversedation. In part for this reason, our patients have probably survived the acute phase only to be saddled with chronic critical illness [14].

Health is characterized by gradual transitions and homeostatic adaptation to a range of stressors, which can be mechanical, microbial, environmental, or biochemical. Disease is characterized by failure to adapt appropriately to such stressors [15]. When successful, the stress response transitions from an acute "exuberant" phase (perhaps with transient overreaction) into a more chronic phase that precedes recovery. Some data argue that the body, although very wise in its responses and adjustments when stresses are modest and sufficient time is available, cannot successfully confront sudden, severe, life-threatening challenges. Evolution simply did not prepare us to do so without assistance. Rather, our evolved responses to severe and life-threatening stressors might not be designed to save the victim, but rather to accelerate his or her demise.

**Figure 2:**
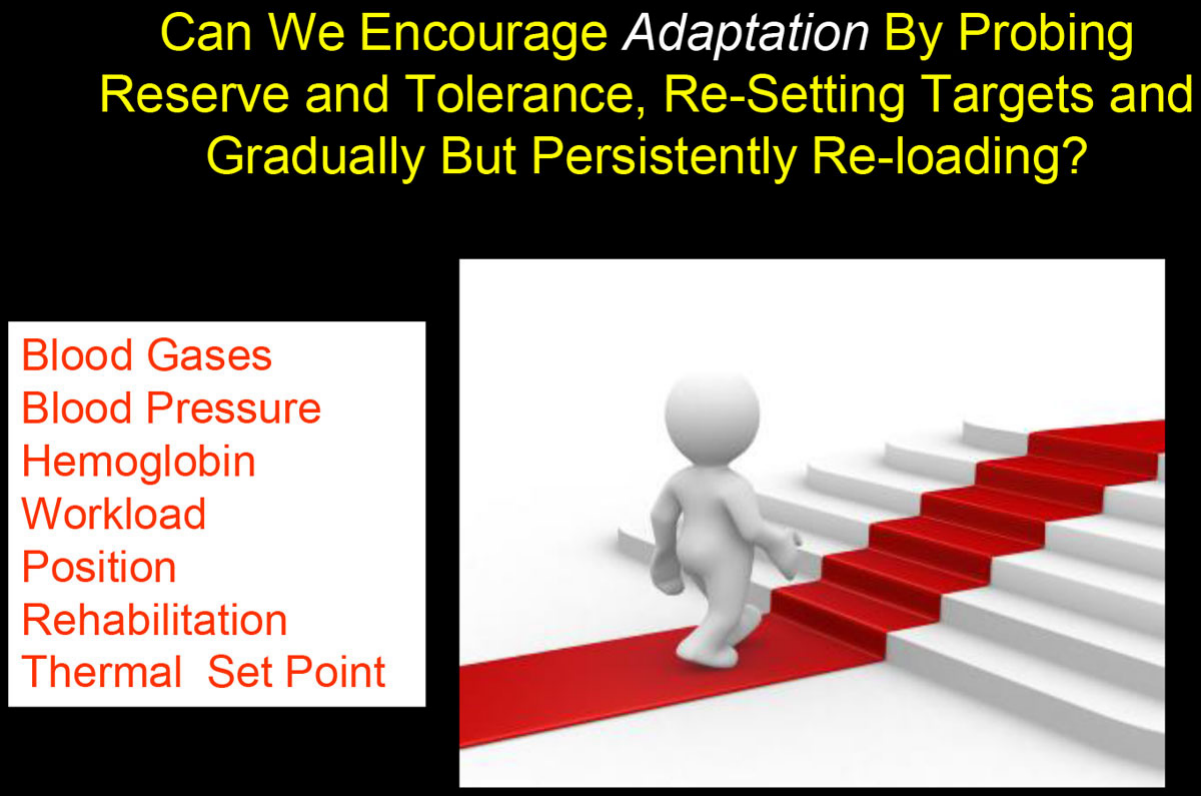
**Potential targets for stepwise adaptation to physiologically abnormal values**.

An ideal physiological response to a disease such as acute respiratory distress syndrome (ARDS) would be to transition from a rescue phase, through a period of stabilization, to graded system reloading and recovery. Whereas rescuing the patient from an overwhelming insult is clearly indicated when natural homeostatic mechanisms are initially destabilized and ineffective, continuing to do so when no longer needed may prevent the patient from adapting to disordered physiology. It is known that, given enough time, the human body is remarkably adaptable during health. This adaptive potential is the basis of athletic conditioning as well as high-altitude acclimatization [15,16]. Examples of how patients adapt to severe disorders of physiology caused by disease can be found in the outpatient setting, as many thrive with remarkable impairments of gas exchange and cardiac function that would disable a healthy patient suddenly confronted with the same deviations from normal parameters. To encourage such adaptation in the varied settings of critical illness will require us to first minimize (buffer) the signal that otherwise stimulates an overly exuberant stress response and then to gradually reload the body so as to encourage successful adaptation (Figure 2). One relatively simple example might be to gradually reduce the inspired oxygen concentration to simulate altitude acclimatization. Eventually, the patient and the physician would accept "permissive hypoxemia".

Thus, to prevent chronic critical illness we may require a two-stage approach to intensive care. In the first "rescue" phase, the excessive native stress response would have to be restrained by minimizing demand and providing near full support. Afterward, gentle transitions to less intense therapy would take place. Here in this second "adaptation" phase, intermittent stresses and rest periods might be linked with ongoing targeted reductions of vital supports (Figure 2). These would include acclimatization, not only to hypoxemia but also to ventilating pressure, vasopressors, inotropes, upright body positioning, muscular exertion, etc.

### Idea

First, shelter the patient from the sudden "storm" at disease onset by restraining the initial stimulus and response to stress. In later days of treatment, condition the patient to "dance in the rain" by encouraging gradual accommodation to abnormal physiology.

## Practice personalized physiological medicine using handheld microscopy at the bedside

Can Ince, PhD

### Background

Many randomized clinical trials in critical care have been informative about the sampled populations, but their results tend to leave clinicians wondering about value, timing, and application of treatments for the individual patient. Current concepts of personalized medicine envision molecular sequencing and analysis and/or the measurement of biomarkers to allow more effective therapies to be selected for the individual patient. Although such approaches may yield benefit, it seems unlikely for the foreseeable future that taking such an approach can effectively provide ongoing management guidance for the continuously changing physiological status of organ function frequently encountered in the critically ill patient. A more physiological approach based on deeper understanding of tissue responses could allow more precise appreciation of the importance of choosing one fluid over another [17] or more direct assessment of the adequacy of blood flow and hemoglobin concentration in providing effective oxygen delivery. At the present time, global markers of perfusion adequacy such as lactate concentration, although helpful, do not give sufficiently detailed information regarding the primary object of therapeutic interest--the status of the cell. Ultimately, a reliable technique for assessing the physiological status of subcellular structures would be at hand to help the clinician select appropriate treatment. This information would then allow an integrative physiological evaluation of hemodynamic coherence between the different physiological compartments in the hierarchy of the cardiovascular system from the systemic circulation down to subcellular organelles in a continuous manner in response to individualized therapy [18].

**Figure 3:**
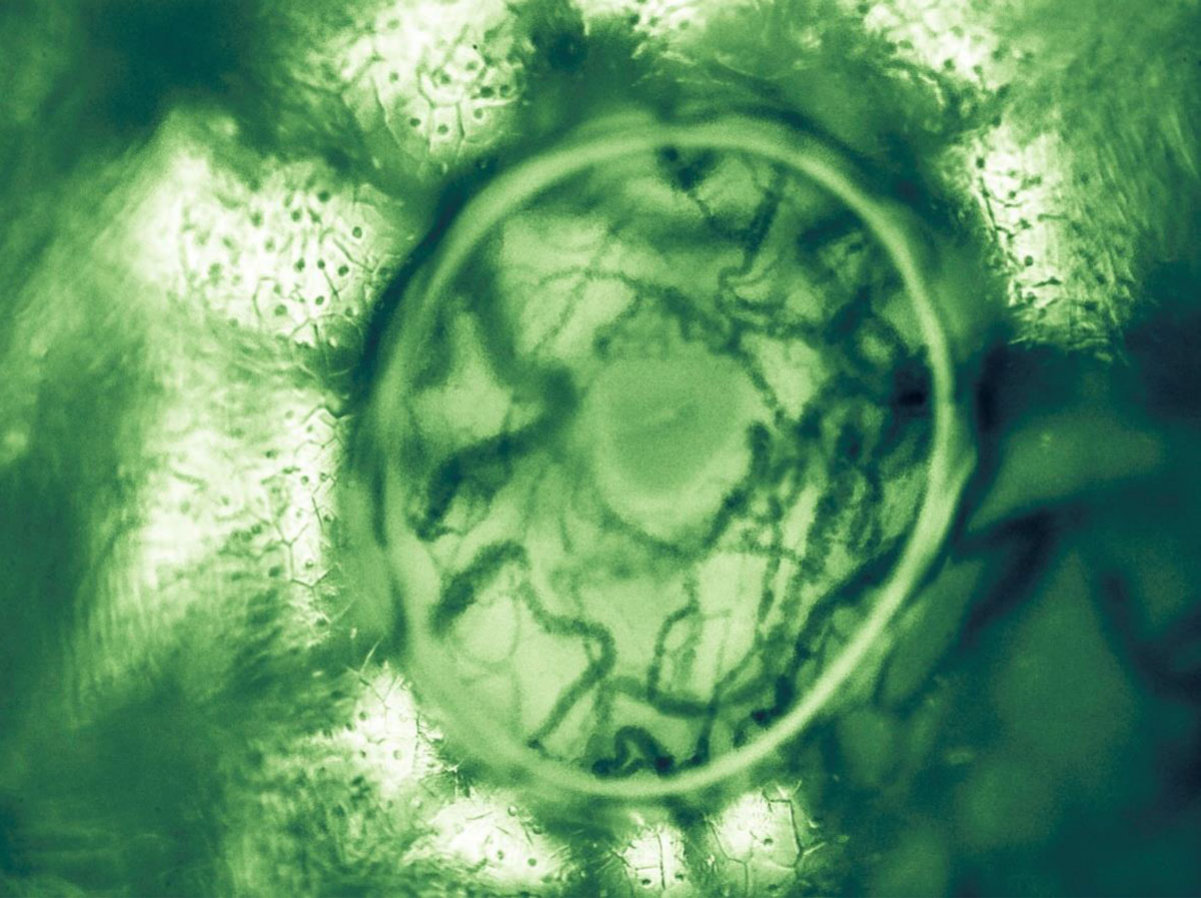
**Sublingual microcirculation image with a trapped bubble showing the microcosm of sublingual vascular and parenchymal cells**. A bubble trapped under the lens cap and provided an optical effect whereby extra magnification and contrast was achieved at the perimeter of the image. Seen in the center is the opening to a submandibular duct surrounded by sublingual microcirculation consisting of red and white blood cells flowing in capillaries and venules.

An alternative approach to genetic testing for accomplishing individualized medicine would be to use personalized physiological medicine to complement the information from genomics or randomized clinical trials. For comprehensive insight into the physiological determinants of disease and/or the effectiveness of treatment, more detailed information is required at the level of the regional microcirculation. Bedside handheld video microscopy of well-selected and accessible "bellwether" tissues may provide essential clinical information in more sensitive fashion than do conventional systemic hemodynamic variables. Such detailed information as leukocyte kinetics, red blood cell sequestration, and even subcellular abnormalities of the endothelial glycocalyx have already been demonstrated with these devices. Improved optics of the latest generation of instruments [19] may allow the frontier to move from the detailed observations of the microcirculation to the function of the individual cell (Figure 3) so as to lay the foundations for truly personalized critical care medicine.

### Idea

Deploy personalized physiological medicine using handheld microscopes that provide detailed, real-time functional observations of the microcirculation, cellular, and subcellular structures. Doing so at the bedside and integrating this information with measures of organ function and the systemic circulation provide the clinician with personalized physiological medicine to confidently apply tailored therapy to meet the true underlying needs of the critically ill patient.

## Abbreviations

ARDS, acute respiratory distress syndrome; CRRT, continuous renal replacement therapy; FCCM, Future of Critical Care Medicine; VILI, ventilator-induced lung injury.

## Competing interests

CI has received honoraria and independent research grants from Fresenius-Kabi, Bad Homburg, Germany; and has developed SDF imaging and is listed as inventor on related patents commercialized by MicroVision Medical under a license from the Academic Medical Center. Braedius Medical, a company owned by a relative of CI, has developed and designed a handheld microscope called CytoCam-IDF imaging. SAK-L has received honoraria for lectures on fluid therapy from B. Braun and Fresenius Kabi; and Fresenius Kabi supported her pooled analysis of randomized clinical trials comparing blood loss in major surgery by covering the costs for biometrical analysis performed by an independent institution. SAK-L received no honorarium and funding for this publication. CP received financial support as research grants and an unrestricted academic research grant, as well as nonrestrictive research grant and consulting fees, from Abbott, Baxter, B. Braun, Cosmed, Fresenius-Kabi, Nestle Medical Nutrition, Novartis, Nutricia-Numico, Pfizer, and Solvay, outside the submitted work. The remaining authors declare that they have no competing interests.
